# It’s About All of Us: Defining and Defending DEI in Gerontological Education

**DOI:** 10.1093/geroni/igaf122.688

**Published:** 2025-12-31

**Authors:** Rona Karasik, Kyoko Kishimoto

**Affiliations:** Saint Cloud State University, Saint Cloud, Minnesota, United States; St. Cloud State University, St. Cloud, Minnesota, United States

## Abstract

This is a critical period in teaching about DEI, where the concepts of diversity, equity and inclusion are under attack, demonized, and purposefully misrepresented for political gain. Now, more than ever, students preparing to work in gerontology and geriatrics need a broad understanding of how policies and events impact groups differently over their life course. This presentation lays the groundwork for demystifying DEI -- what it is, what it does, and why DEI is essential to gerontology and geriatrics education, theory, research, policy and practice. Despite its focus on diminishing discrimination and increasing recognition of historically marginalized groups (e.g., people of color, women, older adults, sexual and gender minorities, the economically and socially disenfranchised), DEI’s scope and purpose are often misunderstood and mistakenly maligned as a zero-sum game such that any effort to obtain basic rights for some necessarily means a loss for others. Rather than recognizing the mutual societal benefit of educational programs, employment opportunities, and research (both medical and social), the current attack on “all things DEI” threatens to erase the hard-won (albeit sorely incomplete) advances made through civil rights actions, the women’s movement, the Americans with Disabilities and Older American’s Acts, socioeconomic and healthcare programs including Social Security, Medicare and Medicaid, and the like. By defining and clarifying the concepts of diversity, equity and inclusion as they relate to gerontological education, this presentation highlights DEI’s connection to people of all ages and backgrounds. Ultimately, DEI isn’t about “them”; it’s about “us” -- all of us.

